# Altered cytokine profiles in relapsed paucibacillary leprosy: a case report

**DOI:** 10.1186/s12879-021-06836-8

**Published:** 2021-11-13

**Authors:** Tomoyuki Sameshima, Yumi Maeda, Tetsu Mukai, Masamichi Goto

**Affiliations:** 1 National Sanatorium Hoshizuka Keiaien, 4204 Hoshizuka-cho, Kanoya, Kagoshima Japan; 2 Leprosy Research Center, National Institute of Infectious Diseases, 4-2-1 Aoba-cho, Higashimurayama, Tokyo Japan; 3 National Hospital Organization Kagoshima Medical Center, 8-1 Shiroyama-cho, Kagoshima, Kagoshima Japan

**Keywords:** Case report, Paucibacillary leprosy, Relapse, Cytokine profiles

## Abstract

**Background:**

Individuals with relapses of leprosy should be monitored carefully, however, with respect to paucibacillary (PB) leprosy, it is sometimes difficult to make a definitive diagnosis of relapse, because the bacillary index is often negative. To evaluate the usefulness of cytokine profiling in a patient with relapsed PB leprosy who tested negative for anti-phenolic glycolipid-I antibodies, we analyzed the *Mycobacterium leprae* protein-induced cytokine expression in peripheral blood mononuclear cells of the patient.

**Case presentation:**

An 89-year-old-male relapsed PB patient, first treated for leprosy over 50 years prior, was examined. In April 2012, he noticed three skin lesions consisting of annular erythema in the thighs. Slit skin smear tests were negative, and skin biopsies revealed a pathology of indeterminate-to-borderline tuberculoid leprosy. He received 600 mg of rifampicin once per month and 75 mg of dapsone daily for 12 months. The annular erythemas disappeared after starting treatment. Before treatment, and 6 and 12 months after starting treatment, the Th1/Th2 cytokine profiles in the supernatant of mononuclear cells from the patient before and after stimulation with *Mycobacterium leprae* soluble protein (MLS) were examined using a Cytometric Bead Array (CBA) Human Th1/Th2 Cytokine Kit II. The CBA Enhanced Sensitivity Flex Set system was applied to detect small amounts of cytokines in the serum just before treatment and one year before relapse. In the culture supernatant, just before treatment, increases in IFN-γ level and the IFN-γ/IL-10 ratio and a decreased IL-6 level were observed without stimulation. Upon stimulation with MLS, just before treatment, both the IFN-γ and TNF levels increased markedly, and twelve months after starting treatment, the IFN-γ and TNF levels decreased greatly. In the serum, just before treatment, increases in IFN-γ and TNF levels and the IFN-γ/IL-10 ratio were evident compared with those measured one year before relapse.

**Conclusions:**

Cytokine profiling using culture supernatants and serum samples may be useful for the diagnosis of relapsed PB leprosy.

## Background

In Japan, the number of new leprosy cases was recently less than 10 per year [1], and the number of relapsed cases per year was also small after the introduction of multidrug therapy (MDT) [2, 3]. For the detection of relapsed cases of leprosy, individuals should be carefully examined, referring to the clinical symptoms, the titres of *Mycobacterium leprae*-specific serum antibodies, slit skin smear tests for *M. leprae* detection, and histopathological examination of skin biopsies. With respect to paucibacillary (PB) leprosy, it might be difficult to make a definitive diagnosis because of the difficulty in demonstrating bacilli in PB cases and the absence of an in vitro cultivation method for* M. leprae*. The criteria for the diagnosis of relapse in PB leprosy depend primarily on clinical features [4]. The World Health Organization has estimated a risk of relapse of 0.77% for MB and 1.07% for PB patients 9 years after stopping MDT [5].

For serodiagnosis of PB leprosy as well as multibacillary (MB) leprosy, a particle agglutination test, Serodia leprae®, has been used to detect anti-phenolic glycolipid-I (PGL-I) IgM antibodies [6]. The seropositivity rates of this agglutination test are reported to be 43% in PB leprosy cases undergoing multidrug therapy [7].

For better understanding of the pathophysiology of relapsed PB leprosy, we examined the immune status of this case. Firstly, when the sera were tested for the presence of anti-PGL-I antibodies, the Serodia leprae result was negative. Secondly, we performed ELISA for the detection of anti-major membrane protein (MMP)-II IgG antibodies, whose positive rate in either treated or untreated PB leprosy cases is greater than that of ELISA for anti-PGL-I IgM antibodies (39.0% vs. 19.5%) [8]. However, again the patient tested negative for anti-MMP-II antibodies. Therefore, we performed a study to evaluate the efficacy of cytokine profiling for the diagnosis of relapsed PB leprosy, examining major cytokines by cytometric bead array (CBA) methods, although several new methods are being developed to detect PB leprosy cases [9].

## Case presentation

An 89-year-old-male relapsed PB patient, who was first treated for leprosy over 50 years prior with chaulmoogra oil, promin and dapsone (diaminodiphenyl sulfone, DDS), was examined.

In December 2004, he complained of facial numbness and received 600 mg of rifampicin (RFP) once per month and 75 mg of dapsone daily for 6 months on suspicion of leprosy relapse. Facial numbness disappeared after these treatments (MDT for the PB leprosy).

**Fig. 1:**
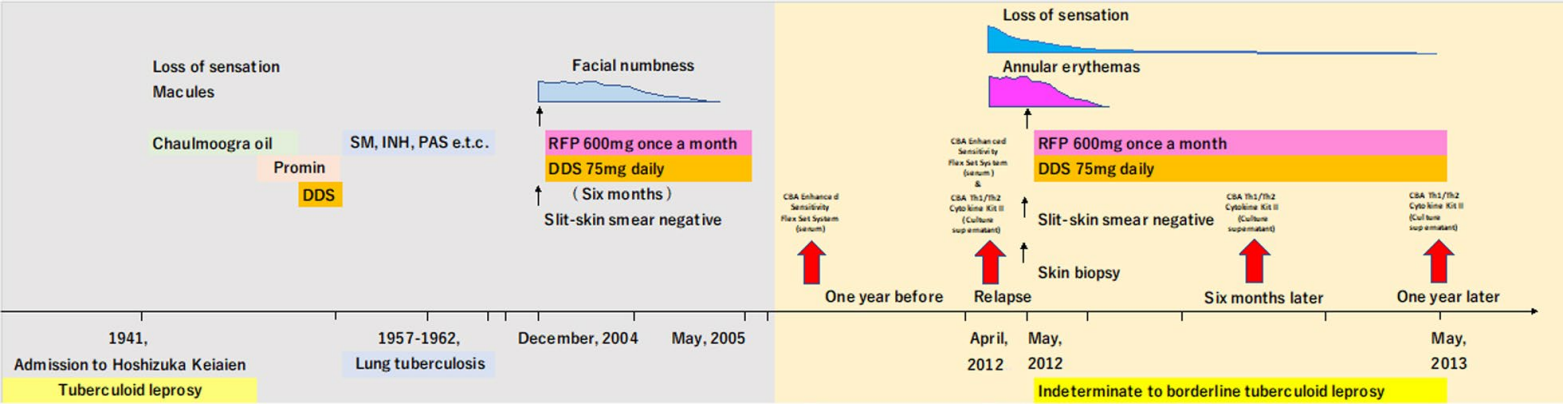
Clinical course of a patient with relapsed PB leprosy. An 89-year-old male relapsed PB patient, who was first treated for leprosy over 50 years prior with chaulmoogra oil, promin and dapsone (diaminodiphenyl sulfone, DDS), was examined. He was diagnosed with lung tuberculosis and treated with streptomycin (SM), isoniazid (INH) and para-aminosalicylic acid (PAS) from 1957 to 1962. In December 2004, he complained of facial numbness and received rifampicin (RFP, 600 mg) once per month and 75 mg of dapsone daily for 6 months. In April 2012, he had three skin lesions, annular erythemas, with loss of sensation in the thighs and again received rifampicin (600 mg) once per month and 75 mg of dapsone daily for 12 months

**Fig. 2:**
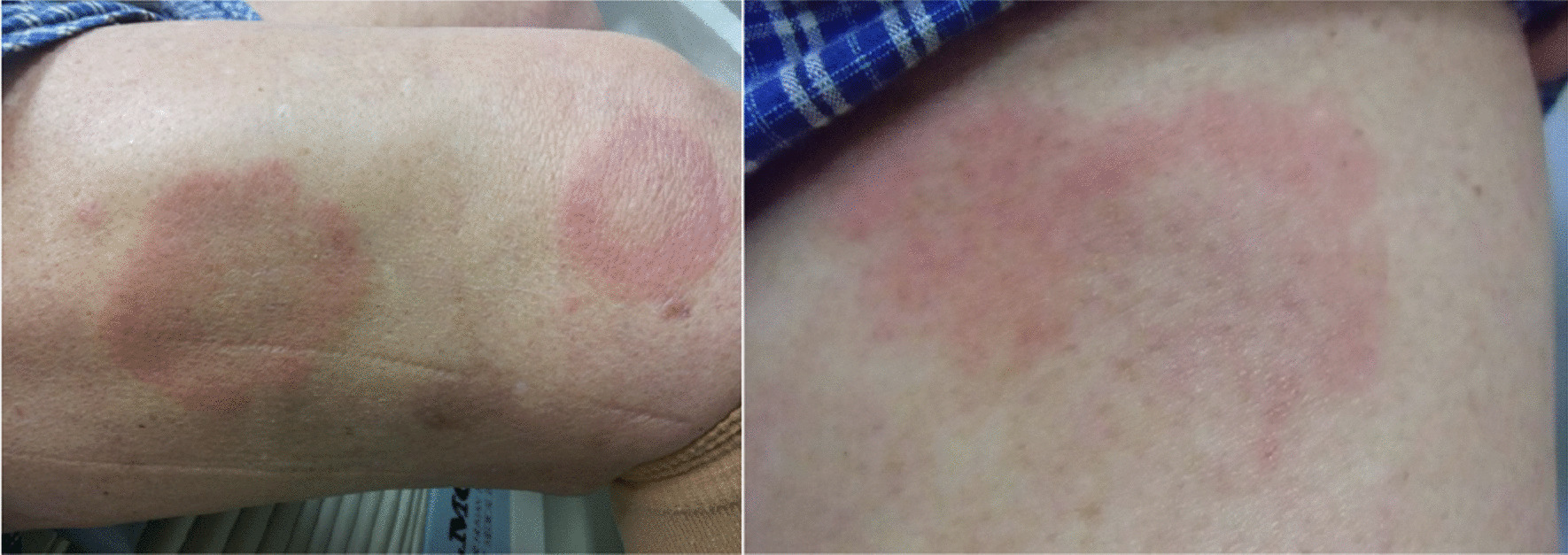
Annular erythemas with loss of sensation in the right (**a**) and left thigh (**b**). These lesions disappeared approximately one month after starting treatment

**Fig. 3:**
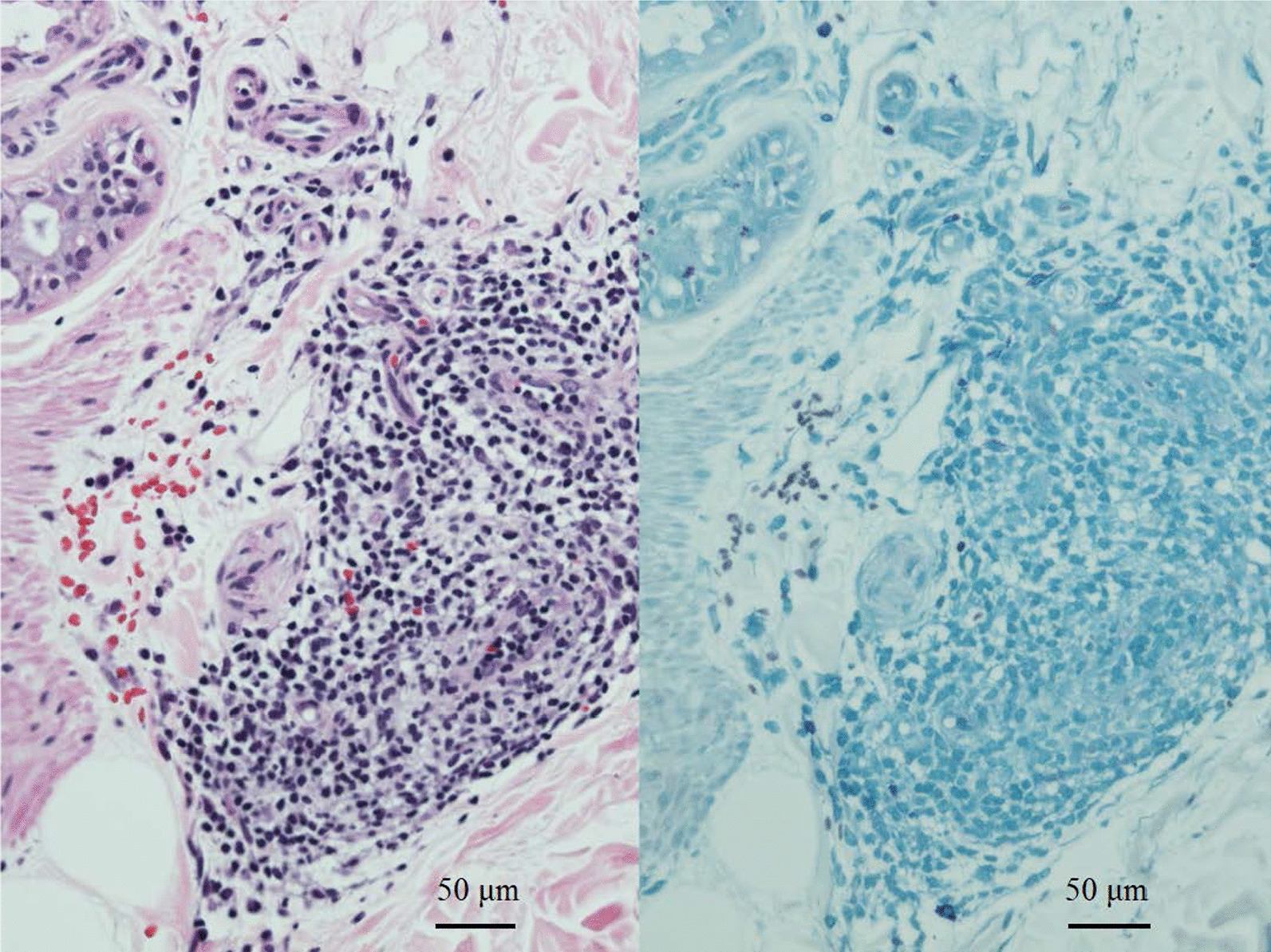
Pathological findings of a skin biopsy. Superficial and deep perineural and perivascular dermatitis, suggestive of early leprosy lesions (I to BT). HE staining (left) and Fite staining (right, no bacilli seen) of a punch biopsy taken from the right thigh. Scale bars measure 50 μm. The microscopy images (magnification × 20) were acquired with the Nikon DS Camera Head DS-Fi1, DS Camera Control Unit DS-L2, and Nikon Eclipse E1000 Microscope at a resolution of 2560 × 1920 pixels

In April 2012, he noticed three skin lesions, annular erythemas, with loss of sensation in the thighs (Figs. 1, 2a, b). Slit skin smear tests were negative and skin biopsies revealed a pathology of indeterminate (I) to borderline tuberculoid (BT) leprosy (Fig. 3). The serum biochemical and haematologic parameters of the patient were almost within the normal range. His body weight was about 62.0 kg and height was about 148.0 cm.

He received 600 mg of rifampicin once per month and 75 mg of dapsone daily for 12 months at the diagnosis of the second relapse of leprosy. The duration of this treatment was extended from 6 to 12 months to improve the therapeutic effect. Approximately one month after starting MDT treatment, the erythemas disappeared and the loss of sensation improved to a nearly normal level of sansation. At the completion of MDT, 12 months after starting treatment, the sensations at healed sites of erythemas on his both thighs were almost normal. Although the patient was an elder, he could understand the duration and efficacy of MDT.

Before treatment and 6 and 12 months after starting treatment, peripheral blood mononuclear cells (PBMCs) were isolated using a BD Vacutainer® CPT Mononuclear Cell Preparation Tube. The Th1/Th2 cytokine profiles (IFN-γ, TNF, IL-2, IL-4, IL-6, and IL-10) in the supernatant of peripheral blood mononuclear cells (PBMCs) from the patient after 96 h of culture were examined using a Cytometric Bead Array (CBA) Human Th1/Th2 Cytokine Kit II. Some mononuclear cells were stimulated with 8 μg/mL *Mycobacterium leprae* soluble protein (MLS) [10], and 2.5 μg/mL phytohemagglutinin (PHA, Sigma cat. No. L1668) was used as the standard stimulant. The MLS was obtained as described previously [10]. Briefly, since *M. leprae* cannot be grown in vitro, *M. leprae* was isolated from the foot pad of *M. leprae*-infected nude mice. The *M. leprae* suspension containing protease inhibitors was mixed with zirconium beads and homogenized. The suspension was centrifuged at 10,000×*g* for 30 min to remove the cell wall debris, and the supernatant was then centrifuged at 100,000×*g*, and the resulting supernatant was considered the *M. leprae* soluble protein (MLS).

Since serum taken from the patient one year before the emergence of the symptoms was available, the CBA Enhanced Sensitivity Flex Set system was applied to detect small amounts of cytokines (IFN-γ, TNF, IL-6, IL-8, IL-10, and IL-17A) in the serum just before treatment and one year before the relapse.

‘The BD™ CBA Human Th1/Th2 Cytokine Kit II can be used to quantitatively measure IL-2, IL-4, IL-6, IL-10, TNF, and IFN-γ protein levels in a single sample. Each capture bead in a BD CBA kit has been conjugated with a specific antibody. The detection reagent provided in the kit is a mixture of phycoerythrin (PE)-conjugated antibodies, which provides a fluorescent signal in proportion to the amount of bound analyte. The individual standard curve range for a given cytokine defines the minimum and maximum quantifiable levels (20 pg/mL and 5000 pg/mL)’ [11]. ‘The BD CBA Human Enhanced Sensitivity Flex Set System employs particles with discrete fluorescence intensities to detect soluble analytes at very low concentrations. The working assay range for most analytes in this system is 274 to 200,000 fg/mL’ [12]. In both systems, when the capture beads and detector reagent are incubated with standards or unknown samples containing recognized analytes, sandwich complexes (capture bead + analyte + detection reagent) are formed. The intensity of PE fluorescence of each sandwich complex reveals the concentration of that particular analyte. These complexes can be measured using flow cytometry to identify particles with fluorescence characteristics of both the bead and the detector. After acquiring samples on a flow cytometer, BD FACSCalibur (dual laser), FCAP Array™ software was used to generate results in graphical and tabular format.

The supernatant of 96 h cultures showed increases in IFN-γ level and the IFN-γ/IL-10 ratio, and a decreased IL-6 level without stimulation, compared with those found after starting treatment. Upon stimulation with MLS just before treatment, both the IFN-γ and TNF levels increased markedly compared with those obtained after six months or one year later. The IL-10 level was lower than the level detected before treatment and increased 6 months after treatment, but returned to undetectable level one year later. The IFN-γ/IL-10 and TNF/IL-10 ratios after stimulation with MLS, six months after starting treatment, were quite high compared with those values obtained with no stimulus. Upon stimulation with MLS, the IFN-γ and TNF levels decreased greatly twelve months after starting treatment, and IL-6 levels increased markedly. IL-2 and IL-4 levels were below the limit of detection (Table 1a, b).

**Table 1:** Cytokine profiles of relapsed PB leprosy in the culture supernatants

	IFN-γ (pg/ml)	TNF (pg/ml)	IL-2 (pg/ml)	IL-4 (pg/ml)	IL-6 (pg/ml)	IL-10 (pg/ml)	IFN-γ/IL-10	TNF/IL-10
*(a) Cytokine profiles of relapsed PB leprosy in the culture supernatants with no stimulus*								
Before treatment (relapse)	6.54	3.22	ND	ND	36.47	1.33	4.91	2.42
Six months later	2.08	2.89	ND	ND	75.25	1.37	1.52	2.11
One year later	1.07	2.96	ND	ND	99.86	0.92	1.16	3.22
*(b) Cytokine profiles of relapsed PB leprosy in the culture supernatants with MLS (8 μg/ml)*								
Before treatment (relapse)	125.16	41.10	ND	ND	17,086.13	ND	N/A	N/A
Six months later	33.07	15.70	ND	ND	13,839.20	0.15	220.47	104.67
One year later	6.04	11.92	ND	ND	16,093.10	ND	N/A	N/A
*(c) Cytokine profiles of relapsed PB leprosy in the culture supernatants with PHA (2.5 μg/ml)*								
Before treatment (relapse)	2915.0	328.3	ND	ND	18,270.9	4.3	676.3	76.2
Six months later	16.5	218.3	ND	ND	17,867.8	5.1	3.2	42.5
One year later	312.5	137.5	ND	ND	18,001.2	4.1	76.4	33.6

96 h cultures, *ND* not detected, below standard range, *N/A* not available

For the stimulation with PHA, the levels of IFN-γ and TNF were similarly higher at relapse than those after the treatment. Slight elevation of IL-10 levels by PHA was observed, so IFN-γ/IL-10 and TNF/IL-10 ratios were calculated, and these values were higher upon relapse. As with MLS, IL-6 levels increased upon stimulation with PHA (Table 1c).

In the serum, just before treatment, increases in IFN-γ and TNF levels and the IFN-γ/IL-10 ratio were evident compared with those measured one year before relapse. The IL-17A level in the serum upon relapse was higher than that in serum collected one year prior (Table 2).

**Table 2:** Serum cytokine profiles of a patient with relapsed PB leprosy

	IFN-γ (pg/ml)	TNF (pg/ml)	IL-6 (pg/ml)	IL-8 (pg/ml)	IL-10 (fg/ml)	IL-17A (fg/ml)	IFN-γ/IL-10	TNF/IL-10
One year prior	1.95	ND	5.70	92.60	533.60*	ND	3.65	N/A
Relapse	10.70	2.23	7.25	93.60	607.00*	409.40*	17.63	3.68

^*^ Cytokines at very low concentrations (fg/ml), *ND* not detected, below standard range, *N/A* not available

## Discussion and conclusions

Paucibacillary (PB) relapse is much more difficult to diagnose since the presentations, including the histological findings, are often indistinguishable from those of a late reversal reaction. Most reversal reactions in PB patients (58/67; 87%) are observed within the first 2 years after discontinuing MDT. In contrast to this, most of registered relapses (27/34; 79%) are observed more than 2 years after discontinuing MDT [13]. The recent symptoms of this patient were not considered to be a reversal reaction, although MDT was administered eight years prior when facial numbness appeared as a symptom of the first leprosy relapse.

Various immunological tests may be useful for monitoring patients on chemotherapy as well as for confirming suspected cases of relapse [4]. The MPLA test using the Serodia leprae kit [6, 7] and dipstick assay [14], both for detection of anti-PGL-I IgM antibodies, have been used as a tool for classification of patients and for identification of those patients who have an increased risk of relapse. As a relatively new serological leprosy test, NDO-LID [15, 16], which is for both anti-PGL-I IgM antibodies and IgG antibodies specific to LID-1 (a synthetic mimetic conjugated to the recombinant fusion protein product of the* M. leprae* genes ML0405 and ML2331), detected larger proportions of multibacillary and paucibacillary leprosy than the alternative, the Standard Diagnostics leprosy test for anti-PGL-I IgM antibodies (87.0% versus 81.7% and 32.3% versus 6.5%, respectively). In paucibacillary leprosy, the seropositivity rates of these tests were not very high, at most, approximately 43%, by the MPLA test during multidrug therapy [7].

When anti-PGL-I IgM antibody levels were examined in either treated or untreated Japanese leprosy patients by ELISA, it was found that only 19.5% of paucibacillary patients showed positive values [8]. The seropositivity rate for anti-major membrane protein(MMP)-II IgG antibodies in the same sera was 39.0%, which was significantly higher than that for anti-PGL-I antibodies [8]. At the time of relapse, the present patient’s serum tested negative with Serodia leprae, which indicated that the patient did not have detectable anti-PGL-I antibodies. Therefore, we performed an ELISA for the detection of anti-MMP-II IgG antibodies. However, the optical density value was also lower than the cut-off value, indicating that the patient tested negative also for anti-MMP-II antibodies.

We then stimulated PBMCs from the patient with MMP-II [17] of *Mycobacterium leprae*, in this occasion, by the same method described above, and the test did not induce effective cytokine production, so we evaluated the production of several cytokines after the stimulation with the *M. leprae* soluble fraction, MLS [10].

In a study in leprosy endemic regions, cytokine multiplex analysis demonstrated significant antigen-specific production of interleukin-1beta (IL-1b), IL-6, and tumor necrosis factor (TNF) in the relapsed multibacillary patients with extremely low IL-10 production, which resulted in a high TNF/IL-10 ratio [18]. In this PB individual, the TNF/IL-10 ratio was rather high upon stimulation with MLS at 6 months after relapse compared with the value obtained with no stimulus. Upon stimulation with MLS just before treatment, if the IL-10 level was calculated although the value was actually lower than the standard range and not detected, the TNF/IL-10 and IFN-γ/IL-10 ratios would be higher than those at six months after relapse. In response to *M. leprae* exposure, the high TNF/IL-10 ratios in tuberculoid patients and the low TNF/IL-10 ratios in household contacts after BCG vaccination are explained to be demonstrating the ways in which IL-10-mediated immunosuppression may act to protect the host from the immunopathological consequences generally associated with the excess of pro-inflammatory cytokines [19].

Since both pro- and anti-inflammatory cytokines play a role in protection from and pathogenesis of mycobacterial diseases, in this respect, their balance, i.e., the IFN-γ/IL-10 ratio, has been described to correlate with potential disease development or response to treatment of tuberculosis or leprosy [20]. In this patient, the IFN-γ/IL-10 ratio was high upon relapse, even without the stimulation with MLS, and decreased in the culture supernatant after treatment. Additionally, in the serum, this ratio was higher at the relapse than one year before the relapse occurred.

Regarding the elevation in IL-6 levels due to stimulation with both MLS and PHA, this phenomenon is considered to be not related to the leprosy relapse. The elevation in IL-10 levels due to stimulation with MLS was not obvious compared with that with stimulation with PHA.

A study found an association of the Th17 response with the PB forms of leprosy [21]. In leprosy reactions, an increase in Th17-associated cytokine levels in the supernatant of antigen stimulated PBMC cultures has been reported [22]. Although the Th17-associated cytokines of this case were not examined in the culture supernatant, the level of IL-17A in the serum at relapse was examined, and its value was significantly elevated compared with the value one year before, even at very low concentrations.

According to the present study, when relapse is suspected in a cured PB leprosy, cytokines such as IFN-γ, TNF and the IFN-γ/IL-10 ratio can be considered to be important markers in the analysis of PBMC culture supernatants. If we could accumulate additional data from the relapsed cases, we might be able to make more reliable diagnoses by referring to the levels of these cytokines or the ratio of cytokines. Using the CBA Enhanced Sensitivity Flex Set system, small amounts of IFN-γ, TNF and IL-10 in the serum were detected more accurately [23, 24]. Comparing the serum cytokine levels with this system before and after the occurrence of relapse may also be helpful for diagnosis.

## Data Availability

The datasets used and/or analyzed during the current study are available from the corresponding author on reasonable request.

## Abbreviations

CBA: Cytometric bead array
ELISA: Enzyme linked immunosorbent assay
TNF: Tumor necrosis factor
IFN: Interferon
IL: Interleukin
MMP: Major membrane protein
MLS: *Mycobacterium leprae* soluble protein
MB: Multibacillary
PB: Paucibacillary
